# Longevity of Humoral Response Six Months Following BNT162b2 Vaccine in Dialysis Patients

**DOI:** 10.3389/fmed.2022.781888

**Published:** 2022-03-25

**Authors:** Timna Agur, Naomi Ben-Dor, Michal Herman-Edelstein, Tali Steinmetz, Shelly Lichtenberg, Shira Schneider, Dafna Yahav, Benaya Rozen-Zvi, Boris Zingerman

**Affiliations:** ^1^Department of Nephrology and Hypertension, Rabin Medical Center, Petah Tikva, Israel; ^2^Sackler Faculty of Medicine, Tel Aviv University, Tel Aviv, Israel; ^3^Infectious Diseases Unit, Rabin Medical Center, Petah Tikva, Israel

**Keywords:** COVID-19, SARS-COV-2, hemodialysis, BNT162b2, anti-spike antibody

## Abstract

**Background:**

End-stage kidney disease substantially increases the risk of severe COVID-19. However, despite early robust immunogenicity of the mRNA-SARS-CoV-2 vaccination in patients with hemodialysis, the longevity of humoral response in this high-risk population is still unknown.

**Methods:**

A prospective cohort study aimed to evaluate the longevity of serologic response in patients with hemodialysis, compared with a control group, 6 months following the second dose of the BNT162b2 vaccine. We assessed antibody response by quantitative measurement of IgG antibodies against the receptor-binding domain of the Spike protein (anti-S1-RBD IgG). Study outcomes were defined as a seropositivity rate and log-transformed anti-S1-RBD IgG levels at 6 months, and the change in antibody levels between 3 and 6 months.

**Findings:**

The cohort included 104 patients with hemodialysis and 84 controls. At a median time of 184 days (IQR, 183–188) following the second dose of the vaccine, 83/104 (79.8%) patients with hemodialysis maintained seropositivity for the anti-S1-RBD IgG level compared to 83/84 (98.8%) in the control group (*p* < 0.001). The log-transformed antibody level was significantly lower in the hemodialysis group (2.23 ± 0.39 log AU/ml vs. 2.69 ± 0.65 log AU/ml, respectively, *p* < 0.001). Older age and hypoalbuminemia were the only variables that were found to be associated with reduced log-transformed antibody levels in univariate and multivariate analysis. There was no interaction between dialysis status and an antibody-level decline rate (*p* = 0.972).

**Conclusion:**

Among patients with hemodialysis, a seropositivity rate and anti-S1-RBD antibody titers were substantially reduced compared with a control group, at 6 months following the second dose of the BNT162b2 vaccine. These findings support the prioritization of patients with hemodialysis for a third “booster” dose.

## Introduction

Patients with an end-stage renal disease requiring maintenance dialysis are a vulnerable population for severe acute respiratory syndrome coronavirus 2 (SARS-CoV-2)-related disease and mortality. Currently, effective vaccines are the only reliable option to mitigate the spread of the COVID-19 pandemic. Hence, it is essential to evaluate the durability of the immune response following vaccination in the high-risk population of hemodialysis (HD) patients (1, 2).

HD Patients have impaired immunity and a diminished response to vaccination (3). The pathogenesis of reduced immunogenicity in HD Patients remained unclear yet is attributed to the combination of the uremic state and multiple comorbidities. Accumulation of uremic toxins and chronic inflammation might contribute to impaired humoral response in the uremic state while advanced age, diabetes, and obesity are among common risk factors that have also been associated with reduced immunity (4–8). Nevertheless, we and other groups have recently shown that HD Patients mount a high final seroconversion rate following the second dose of the BNT162b2 mRNA vaccination, up to 90–95% (9, 10). Yet, from early reports, it seems that the antibody response peak is lower compared with healthy controls (11–13). Whether this early reduced humoral response portends also inferior longevity of the mRNA vaccine immunogenicity is yet to be established (8, 14).

Therefore, we conducted a prospective cohort study aimed to evaluate the longevity of the humoral immune response in HD Patients, compared with a control group, 6 months following the second dose of the BNT162b2 vaccine.

## Methods

### Study Procedures and Data Collection

This was a prospective, single-center, cohort study, evaluating HD patients that had been vaccinated with two doses of BNT162b2 vaccine, 21 days apart. The study was conducted at the two hospital-based dialysis units of RMC (Rabin Medical Center) and was approved by the Ethics Committee of RMC. We included adult patients (>18) receiving both vaccine doses after dialysis treatment initiation. Patients who had documented infection with COVID-19 at any time were excluded from the study. In the first stage of the study, we included 122 HD patients and assessed antibody response about 1 month following the second dose of the vaccine. The settings and primary outcomes have been previously published. In this phase of the study, we further tested consenting participants and control volunteers for SARS-CoV-2 antibody levels at 3 and 6 months after vaccination. Only the HD Patients who had three documented antibody results at 1, 3, and 6 months following the second dose were included. The patients were followed for 1 month after the 6-month antibody test. The study outcomes were defined as a seropositivity rate and log-transformed anti-S1-RBD antibodies at 6 months, and the change in antibody levels between 3 and 6 months.

Clinical, demographic, and laboratory data were obtained by questioning and electronic medical records. Blood samples for SARS-CoV-2 antibodies were collected before the dialysis session. The samples were immediately centrifuged at 3,000 RPM and stored at 4°C. SARS-CoV-2 IgG II Quant (Abbott©) assay was used for quantitative measurement of IgG antibodies against the receptor binding domain of the spike protein (anti-S1-RBD IgG). A positive test was considered if IgG was 50 AU/ml and above, in accordance with the manufacturer’s package insert (15).

### Statistical Analyses

A general linear model was used for comparison between the hemodialysis and the control group, and age, gender, and diabetes were introduced into the model. The estimated marginal mean (EMM) adjusted for the above variables was calculated to evaluate the effect size of dialysis treatment vs. control. Univariate and multivariate linear regression analyses were performed to explore factors associated with a higher log transformed antibody titer. The multivariate model was selected by forward stepwise regression. The results are presented as a change of a log-transformed antibody level per unit of the explanatory variable (*B*).

Matching for age between the study and the control group was done in a 1 to 1 ratio with an age difference of up to 1 year.

To compare between antibody levels at 3- and 6-month time points, we used paired *T*-test and Related-Samples Wilcoxon Signed Rank Test for normally and non-normally distributed variables, respectively. Multivariate repeated measure ANOVA was used to evaluate factors significantly associated with an antibody decline rate between 3 and 6 months.

We evaluated the anti-S1-RBD antibody titer decay rate in the HD cohort group only, who had measured antibody level sampling at three-time points (1, 3, and 6 months after the second dose of the vaccine). A regression line was adjusted to the ln-transformed antibody level because of the expected exponential decay. The estimated time for antibody disappearance was evaluated by the interception point between the regression line and the antibody level of 50 AU/ml (the cut-off of antibody positivity) (16).

## Results

The cohort included 104 HD patients and 84 healthy controls. The average age in the cohort was 73 and 63 years in the control group (*p* < 0.001). Diabetes mellitus (59.6%, 62/114) and male sex (71%, 81/114) were more common among the HD group than the control group (13%, 11/84; 46.4%, 39/84, respectively). Body mass index (BMI) was comparable between the groups (26.8 ± 5.2 vs. 26.6 ± 3.5). Antibody levels were collected at a median time of 184 days (IQR, 183–188) from the second vaccine dose for the study group compared to 203 days (IQR, 196–207) for the control group (Table 1).

**TABLE 1:** Baseline characteristics and antibody response.

Variable	HD	Control	*p-*value
No.	104	84	
Age (years), mean ± SD	73.1 ± 11.6	64 ± 10.9	<0.001
Female gender (%)	33 (31.7%)	45 (53.6%)	0.003
DM (%)	62 (59.6%)	11 (13%)	<0.001
BMI (kg/m^2^), mean ± SD	26.8 ± 5.2	26.6 ± 3.5	0.827
Time since second vaccine dose to 6-month serology median (IQR)	184 (183–188)	203 (196–207)	<0.001
Ab level 6-month (AU/ml) Median (IQR)	147.1 (61.8–433.6)	588.8 (317.6–939.1)	<0.001
Log Ab level 6-month (log AU/ml) mean ± SD	2.23 ± 0.39	2.69 ± 0.65	<0.001
Adjusted log Ab level (EMM) (95% CI)	2.3 (2.19–2.41)	2.63 (2.49–2.77)	0.001
Seronegative (%)	21 (20.2%)	1 (1.2%)	<0.001

*DM, diabetes mellitus; BMI, body mass index; EMM, estimated marginal mean; IQR, interquartile range; CI, confidence interval; SD, standard deviation.*

**FIGURE 1:**
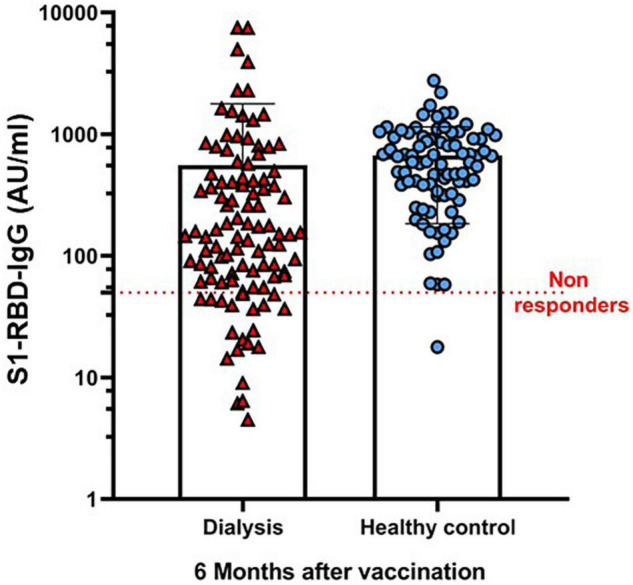
Antibody response at 6 months after the second dose of BNT162b2 vaccine for HD Patients and the healthy control group. Results refer to the HD group (*n* = 104) and the control group (*n* = 84).

Overall, at 6 months after the second dose of the BNT162b2 vaccine, 83/104 (79.8%) HD Patients maintained seropositivity for an anti-S1 antibody level compared to 83/84 (98.8%) in the control group (*p* < 0.001). The median antibody level in the cohort was 147.1 AU/ml (IQR, 61.8–433.6) compared to 588.8 AU/ml (IQR, 317.6–939.13) in the control group (*p* < 0.001). The log-transformed antibody level was also significantly lower in the HD group (2.23 ± 0.39 log AU/ml vs. 2.69 ± 0.65 log AU/ml, respectively, *p* < 0.001) (Table 1 and Figure 1).

After adjustment for age, gender, and diabetes, factors that were different between the cohort and the control groups, the mean of the log-transformed antibody was still significantly lower in the cohort compared to the control group [EMM 2.3 log AU/ml, 95% confidence interval (CI), 2.19–2.41 vs. EMM, 2.63, log AU/ml, 95% CI, 2.49–2.77, respectively] (Table 1).

In an additional age-matched analysis, we included 64 HD Patients and 64 age-matched healthy control participants. The average age in the HD group was 65.9 ± 9.7 years vs. 66.1 ± 9.7 years in the control group (*p* = 0.917). Yet, the log-transformed antibody level at 6 months was significantly lower among the HD group compared with the age-matched control group (2.12 ± 0.86 AU/ml vs. 2.66 ± 0.4 AU/ml, *p* < 0.001).

On univariate and multivariate analyses, the only variables that were associated with lower log-transformed antibody levels among HD Patients were older age [*B*, −0.015 per year, 95% CI (−0.025 to −0.004), *p* = 0.006] and hypoalbuminemia [*B*, −0.459 per year, 95% CI (−0.851 to −0.067), *p* = 0.022] (Table 2).

**TABLE 2:** Factors associated with log-transformed anti-S1-RBD IgG titers in HD Patients.

Variable	Univariate				Multivariate			
	*B* coefficient	95% CI		*p*-value	*B* coefficient	95% CI		*p*-value
Age (per year)	−0.02	−0.03	−0.01	0.004	−0.015	−0.025	−0.004	0.006
Female	−0.04	−0.32	0.23	0.768	–	–	–	–
Dialysis vintage (per month)	0.00	0.00	0.00	0.983	–	–	–	–
DM	0.00	−0.26	0.26	0.975	–	–	–	–
IHD	−0.08	−0.33	0.18	0.548	–	–	–	–
Malignancy Hx	−0.28	−0.59	0.02	0.066	–	–	–	–
Transplantation Hx	−0.29	−0.95	0.37	0.391	–	–	–	–
Dialysis access (jugular catheter vs. AVF)	−0.06	−0.31	0.20	0.664	–	–	–	–
KT/V	0.27	−0.20	0.75	0.259	–	–	–	–
nPCR	0.42	−0.05	0.89	0.077	–	–	–	–
Residual renal function	0.14	−0.11	0.39	0.277	–	–	–	–
BMI (per kg/m^2^)	−0.01	−0.04	0.01	0.403	–	–	–	–
BMI > 30	−0.18	−0.46	0.11	0.222	–	–	–	–
Hemoglobin (per gr/dL)	−0.03	−0.14	0.08	0.609	–	–	–	–
Serum albumin (per gr/dL)	0.46	0.09	0.83	0.015	–	–	–	–
Hypoalbuminemia (Alb < 3.5)	−0.52	−0.92	−0.11	0.012	−0.459	−0.851	−0.067	0.022
Time since second vaccine dose (per day)	0.00	−0.01	0.01	0.001	–	–	–	–
ESA dose (unit per week)	0.00	0.00	0.00	0.802	–	–	–	–
Iron dose (per mg/week)	0.00	0.00	0.00	0.350	–	–	–	–

*B > 0 indicates positive correlation with a log antibody titer. DM, diabetes mellitus; IHD, ischemic heart disease; BMI, body mass index; nPCR, normalized protein catabolic rate; ESA, erythropoietin stimulating agents.*

**FIGURE 2:**
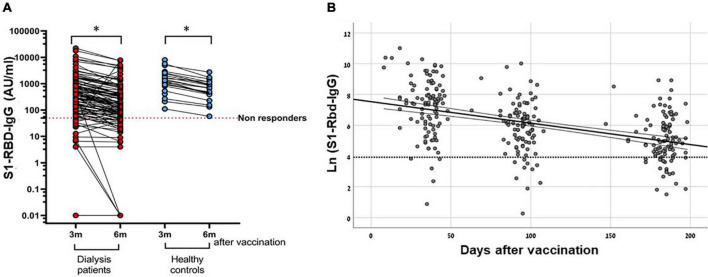
Antibody decay rate in hemodialysis patients following BNT162b2 vaccination. (A) S1-RBD-IgG level 3- and 6-months following 2nd dose of BNT162b2 vaccine in HD patients vs healthy controls. Results refer to HD group (*n* = 104) and control group (*n* = 21). * *P*-value < 0.001 using multivariate repeated ANOVA test. (B) S1-RBD antibody decay rate at 1-, 3- and 6- months following the 2nd dose of BNT162b2 vaccine in HD patients. A regression line was adjusted to the ln-transformed antibody level. Time to Ab level decay below 50 AU/ml - 259.54 days (95% CI 212.98 to 332.17). Results refer to HD group (*n* = 104).

Anti-S1-RBD antibody titers and log-transformed antibody levels at 6 months were significantly reduced, compared to the antibody level at 3 months, in both 104 cohort patients (*p* < 0.001) and 21 control participants (*p* < 0.001), who had documented antibody levels at these two time points (Table 3 and Figure 2A). Comparison of the decline rate between the cohort and control groups did not demonstrate any interaction between dialysis status and an antibody level decline rate (*p* = 0.972).

**TABLE 3:** Anti-S1-RBD IgG levels and log transformed anti-S1-RBD levels at 3 and 6 months for the study and control groups.

		All	*p*-value	HD	*p*-value	Control	*p*-value
No.		125		104		21	

Ab level (AU/ml) median (IQR)	3 months	535.5 (171.8–1,360.2)	<0.001	419.6 (136.6–893.5)	<0.001	1332.5 (915.2–2,437.7)	<0.001
	6 months	217.1 (79.7–687.4)		151 (61.4–423.5)		681.8 (410.9–1,051.3)	

Log transformed Ab level (log AU/ml) mean ± SD	3 months	2.66 ± 0.69	<0.001	2.56 ± 0.71	<0.001	3.09 ± 0.41	<0.001
	6 months	2.34 ± 0.65		2.24 ± 0.67		2.77 ± 0.38	
							

*IQR, interquartile range; CI, confidence interval; SD, standard deviation.*

In an analysis of the anti-S1-R HD patients BD antibody titer decay rate among HD Patients, the estimated time for antibody disappearance (an anti-S1-RBD antibody titer <50 AU/ml) was 259.54 days (95% CI, 212.98–332.17) (Figure 2B).

During a 1-month follow-up period after the 6-month antibody sampling, nine confirmed COVID-19 infections occurred among the cohort group (8.7%). Of them, three were considered a severe disease, and the rest were a mild or asymptomatic disease. Three death events occurred, two were severe COVID-19 disease related, and one was a surgery complication with asymptomatic COVID-19 infection. Only four confirmed COVID-19 infection events in the control group occurred (4.8%). All of them were mild or asymptomatic, with no death event.

In the group of HD Patients, the median Ab level in patients with the mild disease was 459 AU/ml (range, 48.3–2282.3) compared to 51 AU/ml (range, 32.7–67.7) in patients who suffered from severe COVID-19 (*p* = 0.071).

## Discussion

In this cohort of 104 patients with dialysis 6 months following BNT162b2 vaccine, we found that, in contrast to their early humoral response, the long-term seropositivity rate in HD Patients was markedly lower than the seropositivity rate in the control group. The anti-S1-RBD antibody titer substantially declined in both cohort and control. However, while the decay rate was similar in both groups, among the HD group who had a lower antibodies peak at first, the antibody titer was reduced more often below the cutoff value of seropositivity. The estimated time for anti-S1-RBD antibody disappearance was 260 days.

While most of our cohort maintained seropositivity for 6 months, still nine confirmed COVID-19 infection events occurred among the cohort group during a 1-month follow-up period after the 6-month antibody sampling; of them, three were considered severe, whereas only four mild COVID-19 infections occurred in the control group. Notably, during the follow-up month (in August 2021), the administration of a third (booster) dose of BNT162b2 vaccine was offered in Israel for people over 60 years old. Thereby, the documented COVID-19 incidence may not be representative of the natural course of the disease. Intriguingly, despite this major limitation, we found a trend for a correlation between a reduced antibody titer and the risk for severe disease.

At 6 months following vaccination, in univariate and multivariate analysis, risk factors as older age and hypoalbuminemia remained negatively associated with an antibody titer, as we and others have already described (9, 14). Therefore, to validate our results, we adjusted for factors that were significantly different between the cohort and the control groups as age, diabetes, and gender and further added age-matching analysis. Nonetheless, we still documented a substantially lower antibody level among HD patients compared with the control group. These results imply that further to common risk factors, the uremic state *per se* might also be a risk factor associated with impaired humoral response (8).

A recent case series including a small group of 69 patients with dialysis has reported promising results of an early third dose of BNT162b2 up to 13 weeks after the second dose. The third dose improved a seropositivity rate and substantially increased antibody titers in all study patients. Intriguingly, the greatest increase in anti-S1-RBD antibody levels after the third dose has been seen in patients with initial low titers and those with a longer time interval since the second dose (17). Hence, the vulnerable population of patients with dialysis, who demonstrated diminished antibody response after vaccination, might benefit from a delayed booster dose, 6 months after the second dose, which could extend the duration of the vaccine efficacy.

This study has several limitations. It is a single-center observational study. Hence, there is variability in the time between the administration of the second dose and serology, which may be a potential source of bias. We evaluated the durability of the immune response following vaccination by measuring the level of anti-S1-RBD IgG. The importance of waning antibody levels in protecting against disease is still uncertain. We did not assess cellular immunity, nor neutralizing antibodies. Nonetheless, a high correlation has been recently reported between the anti-S1-RBD IgG level and the viral neutralization level (18, 19). Although the correlation between the anti-S1-antibody level and protection against SARS-2-CoV has not yet been unequivocally defined, antibodies are likely to be a major part of the protective response. Furthermore, we assessed antibody response durability of BNT162b2 only and did not evaluate other available COVID-19 vaccines. However, variable immune response to current available COVID-19 vaccines has been documented (10, 20). Monitoring the long-term antibody response may help to define the most effective vaccine, which confers durable and constant immunogenicity in susceptible populations as patients with dialysis.

## Conclusion

In summary, among HD Patients, at 6 months following BNT162b2 vaccine, only 79.8% maintained seropositivity, and anti-S1-RBD IgG titers were substantially reduced compared with a control group. Older age and hypoalbuminemia were independently associated with diminished antibody response. These findings support the prioritization of patients with hemodialysis for a third booster vaccine dose.

## Data Availability Statement

The original data from this study may be available upon reasonable request to the corresponding author.

## Ethics Statement

The studies involving human participants were reviewed and approved by the Rabin Medical Center Ethic Committee. All participants provided their written informed consent to participate in this study.

## Author Contributions

BR-Z, TA, BZ, and NB-D: research design, data collection and analysis, and writing the manuscript. MH-E, TS, SS, SL, and DY: data collection, analysis, and critical review of the manuscript. All authors contributed significantly to this work and approve this manuscript submission.

## Conflict of Interest

The authors declare that the research was conducted in the absence of any commercial or financial relationships that could be construed as a potential conflict of interest.

## Publisher’s Note

All claims expressed in this article are solely those of the authors and do not necessarily represent those of their affiliated organizations, or those of the publisher, the editors and the reviewers. Any product that may be evaluated in this article, or claim that may be made by its manufacturer, is not guaranteed or endorsed by the publisher.
